# GLIM criteria for the diagnosis of malnutrition – A consensus report from the global clinical nutrition community*

**DOI:** 10.1002/jcsm.12383

**Published:** 2019-03-28

**Authors:** T. Cederholm, G.L. Jensen, M.I.T.D. Correia, M.C. Gonzalez, R. Fukushima, T. Higashiguchi, G. Baptista, R. Barazzoni, R. Blaauw, A.J.S. Coats, A.N. Crivelli, D.C. Evans, L. Gramlich, V. Fuchs‐Tarlovsky, H. Keller, L. Llido, A. Malone, K.M. Mogensen, J.E. Morley, M. Muscaritoli, I. Nyulasi, M. Pirlich, V. Pisprasert, M.A.E. de van der Schueren, S. Siltharm, P. Singer, K. Tappenden, N. Velasco, D. Waitzberg, P. Yamwong, J. Yu, A. Van Gossum, C. Compher, GLIM Core Leadership Committee, GLIM Working Group

**Affiliations:** ^1^ Department of Public Health and Caring Sciences, Clinical Nutrition and Metabolism Uppsala University Uppsala Sweden; ^2^ Theme Aging Karolinska University Hospital Stockholm Sweden; ^3^ Dean's Office and Department of Medicine, Larner College of Medicine University of Vermont Burlington VT USA; ^4^ Department of Surgery Universidade Federal de Minas Gerais Belo Horizante Brazil; ^5^ Post‐graduate Program in Health and Behavior Catholic University of Pelotas RS Brazil; ^6^ Department of Medicine, Department of Surgery Tokyo University School of Medicine Tokyo Japan; ^7^ Department of Surgery and Palliative Medicine Fujita Health University School of Medicine Dengakugakubo, Kutsukake Toyoake‐City Aichi Japan; ^8^ Medicine Faculty Central University of Venezuela Universitary Hospital of Caracas, Chief Nutritional Support Unit Hospital Universitary/Academic of Caracas, University Central of Venezuela Venezuela; ^9^ Department of Medical, Technological and Translational Sciences University of Trieste, Ospedale di Cattinara Trieste Italy; ^10^ Division of Human Nutrition, Faculty of Medicine and Health Sciences Stellenbosch University Cape Town South Africa; ^11^ Monash University Australia; ^12^ University of Warwick Warwick UK; ^13^ Unit of Nutrition Support and Malabsorptive Diseases Hospital HIGA San Martín Buenos Aires Argentina; ^14^ Department of Surgery The Ohio State University Columbus OH USA; ^15^ University of Alberta Edmonton Alberta Canada; ^16^ Clinical Nutrition Department Hospital General de México Mexico City Mexico; ^17^ Schlegel‐UW Research Institute for Aging and Department of Kinesiology University of Waterloo Ontario Canada; ^18^ Clinical Nutrition Service St. Luke's Medical Center‐Quezon City Metro‐Manila, Quezon City Philippines; ^19^ The American Society for Parenteral and Enteral Nutrition Silver Spring MD USA; ^20^ Mt. Carmel West Hospital Columbus OH USA; ^21^ Department of Nutrition Brigham and Women's Hospital Boston MA USA; ^22^ Division of Geriatrics Saint Louis University Hospital St. Louis MO USA; ^23^ Department of Clinical Medicine Sapienza University of Rome Italy; ^24^ Department of Nutrition, Alfred Health and Professor of Dietetic Practice, Department of Rehabilitation, Nutrition and Sport, Latrobe University; Department of Medicine, Central Clinical School Monash University Australia; ^25^ Imperial Oak Outpatient Clinic, Endocrinology, Gastroenterology and Clinical Nutrition Berlin Germany; ^26^ Department of Medicine Khon Kaen University College of Medicine Khon Kaen Thailand; ^27^ Department of Nutrition and Dietetics Amsterdam UMC, Vrije Universiteit Amsterdam Amsterdam the Netherlands; ^28^ Faculty of Health and Social Studies, Department of Nutrition and Dietetics HAN University of Applied Sciences Nijmegen the Netherlands; ^29^ Ministry of Science and Technology Bangkok Thailand; ^30^ Department of General Intensive Care Rabin Medical Center Petah Tikva Israel; ^31^ Sackler School of Medicine Tel Aviv University Israel; ^32^ Department of Kinesiology and Nutrition University of Illinois‐Chicago Chicago IL USA; ^33^ Department of Nutrition, Diabetes and Metabolismo, School of Medicine Pontificia Universidad Catolica de Chile Chile; ^34^ Department of Gastroenterology, School of Medicine University of São Paulo São Paulo Brazil; ^35^ Department of Medicine Siriaj Hospital Bangkok Thailand; ^36^ GI Surgery and Nutrition Metabolic Division, Department of General Surgery Peking Union Medical College Hospital Beijing China; ^37^ Department of Gastroenterology, Clinic of Intestinal Diseases and Nutritional Support Hopital Erasme, Free University of Brussels Brussels Belgium; ^38^ Biobehavioral Health Sciences Department and Nutrition Programs University of Pennsylvania School of Nursing Philadelphia PA USA

**Keywords:** Malnutrition, Screening, Assessment, Diagnosis

## Abstract

**Rationale:**

This initiative is focused on building a global consensus around core diagnostic criteria for malnutrition in adults in clinical settings.

**Methods:**

In January 2016, the Global Leadership Initiative on Malnutrition (GLIM) was convened by several of the major global clinical nutrition societies. GLIM appointed a core leadership committee and a supporting working group with representatives bringing additional global diversity and expertise. Empirical consensus was reached through a series of face‐to‐face meetings, telephone conferences, and e‐mail communications.

**Results:**

A two‐step approach for the malnutrition diagnosis was selected, i.e., first screening to identify “at risk” status by the use of any validated screening tool, and second, assessment for diagnosis and grading the severity of malnutrition. The malnutrition criteria for consideration were retrieved from existing approaches for screening and assessment. Potential criteria were subjected to a ballot among the GLIM core and supporting working group members. The top five ranked criteria included three phenotypic criteria (weight loss, low body mass index, and reduced muscle mass) and two etiologic criteria (reduced food intake or assimilation, and inflammation or disease burden). To diagnose malnutrition at least one phenotypic criterion and one etiologic criterion should be present. Phenotypic metrics for grading severity as Stage 1 (moderate) and Stage 2 (severe) malnutrition are proposed. It is recommended that the etiologic criteria be used to guide intervention and anticipated outcomes. The recommended approach supports classification of malnutrition into four etiology‐related diagnosis categories.

**Conclusion:**

A consensus scheme for diagnosing malnutrition in adults in clinical settings on a global scale is proposed. Next steps are to secure further collaboration and endorsements from leading nutrition professional societies, to identify overlaps with syndromes like cachexia and sarcopenia, and to promote dissemination, validation studies, and feedback. The diagnostic construct should be re‐considered every 3–5 years.

## 1 Introduction

Malnutrition due to disease, poverty, hunger, war, and natural catastrophe is a fate suffered by greater than 1 billion of the world's population. Historically, starvation and famine were prevalent causes of malnutrition and they remain so today. However, with improvements in agriculture, education, public health, healthcare, and living standards, nutrition disorders and related conditions now encompass the full scope of undernutrition, micronutrient abnormalities, obesity, cachexia, sarcopenia, and frailty.^1^, ^2^

Malnutrition, e.g. undernutrition, may be caused by compromised intake or assimilation of nutrients but there is growing appreciation that malnutrition may also be caused by disease‐associated inflammatory or other mechanisms. The malnutrition that is associated with disease or injury invariably consists of a combination of reduced food intake or assimilation and varying degrees of acute or chronic inflammation, leading to altered body composition and diminished biological function.^1^, ^2^, ^3^ Inflammation contributes to malnutrition through associated anorexia and decreased food intake as well as altered metabolism with elevation of resting energy expenditure and increased muscle catabolism. Altered body composition manifests as a decrease in any marker of muscle mass (fat‐free mass, muscle mass index or body cell mass). Thus, malnutrition is associated with adverse functional and clinical outcomes.

Although malnutrition is a global concern associated with incremental morbidity, mortality, and cost, there has been a fundamental lack of consensus on diagnostic criteria for application in clinical settings. No single existing approach has secured broad global acceptance.^1^, ^4^, ^5^, ^6^, ^7^, ^8^ Our evolving understanding of the contributions of disease/inflammation may render some concepts of malnutrition in the current International Classifications of Diseases (ICD‐10) (http://www.who.int/classifications/icd/en/) inconsistent with approaches or nomenclature that are currently used in clinical practice and research. Thus, there is an urgent need to establish a global consensus to be used in clinical care settings for adults.

In order to respond to the needs of the clinical nutrition and medical communities the Global Leadership Initiative on Malnutrition (GLIM) was convened in January 2016. GLIM has engaged several of the clinical nutrition societies with global reach to focus on standardizing the clinical practice of malnutrition diagnosis. We also sought to clarify overlaps with related disease classifications including cachexia. The purpose of this specific initiative is to reach global consensus on the identification and endorsement of criteria for the diagnosis of malnutrition in clinical settings.

## 2 Methods

### 2.1 The consensus procedure

On January 19, 2016 the Global Leadership Conversation: Addressing Malnutrition was held at the ASPEN Conference.^9^ Key breakthroughs at that meeting led to the development of GLIM:

1. It was recognized that there was considerable consensus among stakeholders around many malnutrition diagnosis issues
2. There was strong commitment for reaching broader global consensus in defining and characterizing malnutrition
3. A core leadership committee with representatives of several of the global clinical nutrition societies; ASPEN (www.nutritioncare.org), ESPEN (www.espen.org), FELANPE (www.felanpeweb.org) and PENSA (www.pensa‐online.org) was constituted to form GLIM. The core GLIM leadership committee then created a larger supporting working group comprised of invited members that brought additional global diversity and expertise to the consensus effort.
4. It was agreed that a series of face‐to‐face meetings, telephone conferences, and email communications would be used to delineate the GLIM approach.

The first full meeting of the GLIM extended working group was held September 19, 2016 at the ESPEN Congress.^10^ Highlighted objectives included consensus development of evidence‐based criteria suitable to diverse clinical settings, global dissemination of consensus criteria, and the priority to seek adoption by leading diagnosis classification and coding entities across the globe. It was also agreed that the desired approach to malnutrition diagnosis should be simple and include clinically relevant diagnostic criteria that will be appropriate for application by all healthcare professionals using methods that are widely available. The intent was also to promote global use of consensus criteria that can be readily used with other approaches and additional criteria of regional preference.

## 3 Results

Consensus was gradually achieved over the course of the GLIM meetings held February 20, 2017 at the ASPEN Conference,^11^ September 11, 2017 at the ESPEN Congress, and January 25, 2018 at the ASPEN Conference. Meanwhile, discussions were also held with the leadership of The Society of Sarcopenia, Cachexia and Wasting Disorders (SCWD).

### 3.1 A two‐step model for risk screening and diagnosis assessment

There was strong consensus that the key first step in the evaluation of nutritional status is malnutrition risk screening to identify “at risk” status by the use of any validated screening tool^12^, ^13^, ^14^; some of these tools are noted in Table 1 and the Appendix. This is followed by the second step of assessment for diagnosis and severity grading as described below.

**Table 1:** Survey of existing approaches used in screening and assessment of malnutrition and cachexia.

	**NRS‐2002** 12, a	**MNA‐SF** 21, a ^,^ b	**MUST** 22, a	**ESPEN 2015** 8, a	**ASPEN/AND** 7, a	**SGA** 4, a	**Evans 2008** 5, c	**PEW 2008** 23, d	**Fearon 2011** 6, c
***Etiologies***									
Reduced food intake	X	X	X	X	X	X		X	X
Disease burden/inflammation	X	X	X	X	X	X	X	X	X
***Symptoms***									
Anorexia		X				X	X		X
Weakness		X				X	X		
									
***Signs/Phenotype***									
Weight loss	X	X	X	X	X	X	X	X	X
Body mass index	X	X	X	X			X	X	X
Lean/fat free/muscle mass		X		X	X	X	X	X	X
Fat mass					X	X		X	
Fluid retention/ascites					X	X			
Muscle function; e.g. grip strength					X	X	X		
Biochemistry							X	X	

NRS‐2002: Nutritional Risk Screening‐2002, MNA‐SF = Mini Nutritional Assessment‐Short Form, MUST = Malnutrition Universal Screening Tool, ESPEN = European Society for Clinical Nutrition and Metabolism, ASPEN = American Society of Parenteral and Enteral Nutrition, AND = Academy of Nutritiona and Dietetics, SGA = Subjective Global Assessment, PEW=Protein Energy Wasting

a Malnutrition approach

b Adapted for older adults

c Cachexia approach

d Adapted for chronic kidney disease

### 3.2 Criteria selected for malnutrition diagnosis

A comprehensive survey of existing approaches used in screening and assessment of malnutrition was conducted to identify criteria worthy of consideration (Table 1 and the Appendix). It was recognized that these approaches incorporate multiple common criteria. For example, the presence of weight loss and disease burden or inflammation is common to most of them as is reduced food intake (Table 1). Potential consensus criteria from existing approaches as well as additional criteria suggested by participants were subject to further consideration.

In order to establish consensus and endorsement of a minimum set of diagnostic criteria by the core leadership committee and the supporting working group a formal ballot was administered whereby participants ranked proposed diagnosis criteria. The top 5 ranked criteria by an overwhelming majority of GLIM participants were as follows:

- Weight loss
- Low body mass index (BMI)
- Reduced muscle mass
- Reduced food intake or assimilation
- Disease burden/inflammation

### 3.3 Weight loss

There was strong GLIM consensus for the inclusion of weight loss as a phenotypic criterion. Validity is well established and there is a robust literature on which threshold selection could be based (Appendix). There must be priority to obtain repeated weight measures over time to identify trajectories of decline, maintenance, and improvement. GLIM participants felt that it is especially important to recognize the pace of weight loss early in the course of disease or injury and to highlight that many patients will have lost appreciable weight prior to presenting to the healthcare.

### 3.4 Low BMI

There is substantial regional variation in the use of low BMI as a phenotypic criterion for malnutrition diagnosis. North American GLIM representatives indicated that low BMI is seldom used as a clinical malnutrition marker in those regions. The experience from the current American population is that people are often overweight or obese and would need to lose substantial weight before low BMI designation would occur. Since other regions of the world currently make use of BMI as a criterion for recognition of malnutrition, the GLIM consensus includes low BMI. Further research is however needed to secure consensus reference BMI data for Asian populations in clinical settings.

### 3.5 Reduced muscle mass

Reduced muscle mass is a phenotypic criterion with strong evidence to support its inclusion in the GLIM consensus criteria. However, there is not consensus regarding how best to measure and define reduced muscle mass, particularly in clinical settings. Therefore, GLIM recommends measurement by dual‐energy absorptiometry or other validated body composition measures such as bioelectrical impedance, ultrasound, computed tomography or magnetic resonance imaging, but these methods are still not available in most settings for nutritional assessment throughout the globe. Physical examination or anthropometric measures of calf or arm muscle circumference are therefore included as alternative measures. Recommendations are likely to evolve as portable and less costly body composition technologies are developed and become widely available.

For the purpose of recommended cut‐off values for muscle mass reductions, GLIM refers to recommendations from the European Working Group on Sarcopenia in Older People (EWGSOP)^15^ and from The Foundation of National Institute of Health (FNIH) initiative,^16^ and the Asian Working Group on Sarcopenia (AWGS).^17^ Reference standards for muscle mass may warrant adjustment for race and sex. Additional research is warranted to establish general reference standards as well as for some specific populations, e.g. in Asia. Examples of recommended thresholds are found in Table 2.

**Table 2:** Examples of recommended thresholds for reduced muscle mass

	Males	Females
Appendicular Skeletal Muscle Index (ASMI, kg/m^2^)^15^	<7.26	<5.25
ASMI, kg/m^2^ 24, 1	<7	<6
ASMI, kg/m^2^ 17, 2		
‐ DXA	<7	<5.4
‐ BIA	<7	<5.7
Fat free mass index (FFMI, kg/m^2^)^8^	<17	<15
Appendicular lean mass (ALM, kg)^25^	<21.4	<14.1
Appendicular lean mass adjusted for BMI = ALM/BMI^26^	<0.725	<0.591

DXA = dual energy x‐ray absorptiometry, BIA = bioelectrical impedance analysis

BMI = body mass index

1 Recommendations from European Working Group on Sarcopenia in Older People 2 (EWGSOP2); personal communication Alfonso Cruz‐Jentoft.

2 Recommendations from Asian Working Group for Sarcopenia (AWGS) for Asians.

Assessment of muscle function using grip strength or other validated procedures is recommended as a supportive measure in the GLIM consensus (Tables 3 and 4). Decline in muscle strength generally exceeds changes in muscle size.^18^ However, irrespective of etiology, appreciable loss of muscle mass is generally accompanied by reduced muscle function. In situations where muscle mass cannot be readily assessed then muscle strength, e.g. hand grip strength, is an appropriate supporting proxy.

**Table 3:** Phenotypic and etiologic criteria for the diagnosis of malnutrition.

**Phenotypic Criteria** *			**Etiologic Criteria** *	
**Weight loss (%)**	**Low body mass index (kg/m** ^**2**^ **)**	**Reduced muscle mass** a	**Reduced food intake or assimilation** b ^**,**^ c	**Inflammation** d ^**,**^ e ^**,**^ f
>5% within past 6 months, or>10% beyond 6 months	<20 if <70 years, or <22 if >70 years	Reduced by validated body composition measuring techniques^a^	≤50% of ER >1 week, or any reduction for >2 weeks, or any chronic GI condition that adversely impacts food assimilation or absorption^b^^,^ c	Acute disease/injury^d^^,^ f or chronic disease‐related^e^^,^ f
	Asia: <18.5 if <70 years, or <20 if >70 years			

* Requires at least 1 phenotypic criterion and 1 etiologic criterion for diagnosis of malnutrition.

a For example fat free mass index (FFMI, kg/m^2^)) by dual‐energy absorptiometry (DXA) or corresponding standards using other body composition methods like bioelectrical impedance analysis (BIA), CT or MRI. When not available or by regional preference, physical examination or standard anthropometric measures like mid‐arm muscle or calf circumferences may be used. Thresholds for reduced muscle mass need to be adapted to race (Asia). Functional assessments like hand‐grip strength may be considered as a supportive measure.

b Consider gastrointestinal symptoms as supportive indicators that can impair food intake or absorption e.g. dysphagia, nausea, vomiting, diarrhea, constipation or abdominal pain. Use clinical judgement to discern severity based upon the degree to which intake or absorption are impaired. Symptom intensity, frequency, and duration should be noted.

c Reduced assimilation of food/nutrients is associated with malabsorptive disorders like short bowel syndrome, pancreatic insufficiency and after bariatric surgery. It is also associated with disorders like esophageal strictures, gastroparesis, and intestinal pseudo‐obstruction. Malabsorption is a clinical diagnosis manifest as chronic diarrhea or steatorrhea. Malabsorption in those with ostomies is evidenced by elevated volumes of output. Use clinical judgement or additional evaluation to discern severity based upon frequency, duration, and quantitation of fecal fat and/or volume of losses.

d Acute disease/injury‐related. Severe inflammation is likely to be associated with major infection, burns, trauma or closed head injury. Other acute disease/injury‐related conditions are likely to be associated with mild to moderate inflammation.

e Chronic disease‐related. Severe inflammation is not generally associated with chronic disease conditions. Chronic or recurrent mild to moderate inflammation is likely to be associated with malignant disease, chronic obstructive pulmonary disease, congestive heart failure, chronic renal disease or any disease with chronic or recurrent Inflammation. Note that transient inflammation of a mild degree does not meet the threshold for this etiologic criterion.

f C‐reactive protein may be used as a supportive laboratory measure.

GI = gastro‐intestinal, ER = energy requirements

**Table 4:** Thresholds for severity grading of malnutrition into stage 1 (moderate) and stage 2 (severe) malnutrition

	**Phenotypic Criteria** a		
	**Weight loss (%)**	**Low body mass index (kg/m** ^**2**^ **)** b	**Reduced muscle mass** c
**Stage 1/Moderate Malnutrition** (Requires 1 phenotypic criterion that meets this grade)	5–10% within the past 6 mo, or 10–20% beyond 6 mo	<20 if <70 yr, <22 if ≥70 yr	Mild to moderate deficit (per validated assessment methods – see below)
**Stage 2/Severe Malnutrition** (Requires 1 phenotypic criterion that meets this grade)	>10% within the past 6 mo, or >20% beyond 6 mo	<18.5 if <70 yr, <20 if ≥70 yr	Severe deficit (per validated assessment methods – see below)

a Severity grading is based upon the noted phenotypic criteria while the etiologic criteria described in the text and Figure 1 are used to provide the context to guide intervention and anticipated outcomes.

b Further research is needed to secure consensus reference BMI data for Asian populations in clinical settings.

c For example appendicular lean mass index (ALMI, kg/m^2^) by dual‐energy absorptiometry or corresponding standards using other body composition methods like bioelectrical impedance analysis (BIA), CT or MRI. When not available or by regional preference, physical examination or standard anthropometric measures like mid‐arm muscle or calf circumferences may be used. Functional assessments like hand‐grip strength may be used as a supportive measure.^15^

### 3.6 Reduced food intake or assimilation

Reduced food intake is a well‐established etiologic criterion for malnutrition that has strong validity. It can have multiple causes including poor oral health, medication side effects, depression, dysphagia, gastrointestinal complaints, anorexia and inadequate nutrition support. Thresholds for relevant impairment of food intake are widely reported (Appendix) and GLIM participants sought to empirically provide a practical synthesis. Reduced assimilation of food/nutrients is associated with malabsorptive disorders like short bowel syndrome, pancreatic insufficiency and after bariatric surgery. It is also associated with disorders like esophageal strictures, gastroparesis, and intestinal pseudo‐obstruction, as well as with gastrointestinal symptoms like dysphagia, nausea, vomiting, diarrhea, constipation, and abdominal pain. These symptoms have been incorporated as supportive indicators into this GLIM consensus criterion to help to identify poor food intake or assimilation.

### 3.7 Disease burden/inflammation

GLIM members recognized that disease burden/inflammation has become a widely accepted etiologic criterion in existing screening and assessment tools (Table 1). Clinical diagnosis provides a simple approach to recognition of severe, chronic or frequently recurrent inflammation.^1^, ^2^, ^19^ For example, major infections, burns, trauma, and closed head injury are associated with acute inflammation of a severe degree. Indicators of inflammation may include fever, negative nitrogen balance, and elevated resting energy expenditure. Most chronic organ diseases, like congestive heart failure, chronic obstructive pulmonary disease, rheumatoid arthritis, chronic kidney or liver disease and cancer, are associated with chronic or recurrent inflammation of a mild to moderate degree. While severe inflammation is generally easy to discern, clinical judgement is often required to recognize that of lesser degree. Supportive proxy measures of inflammation can include laboratory indicators like serum C‐reactive protein (CRP), albumin, or pre‐albumin.

### 3.8 Approach to using combined phenotypic and etiologic criteria for malnutrition diagnosis

Weight loss, reduced BMI, and reduced muscle mass were categorized as phenotypic criteria, and reduced food intake/assimilation and disease burden/inflammation as etiologic criteria (Table 3 and Figure 1). For the diagnosis of malnutrition, GLIM recommends that the combination of at least one phenotypic criterion and one etiologic criterion is required (Table 3 and Figure 1). The selection of threshold values for the consensus diagnostic criteria was guided by review of existing approaches used in screening and assessment as was the selection of threshold values for severity grading described below (see Appendix). The selected threshold values for diagnosis of malnutrition are shown in Table 3. While only the phenotypic criteria are proposed for the severity grading that follows, the inclusion of the etiologic criteria for malnutrition diagnosis is deemed a priority to guide appropriate intervention and anticipated outcomes.

**Figure 1:**
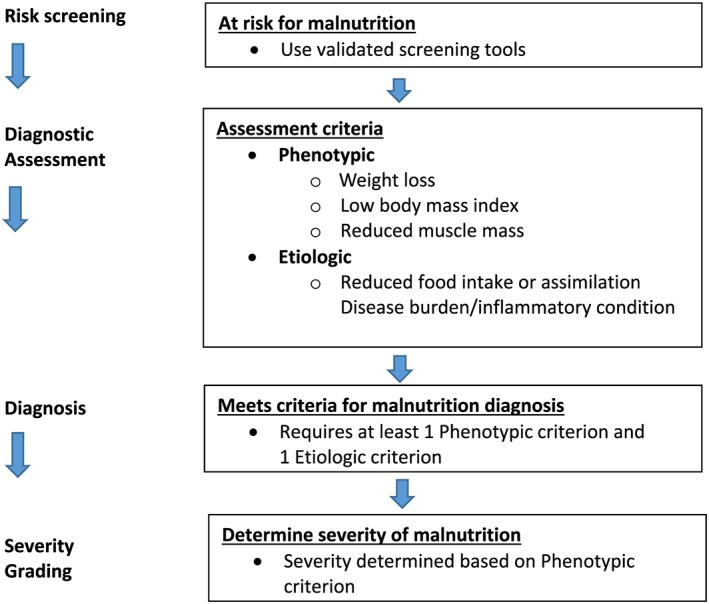
GLIM diagnostic scheme for screening, assessment, diagnosis and grading of malnutrition.

### 3.9 Severity grading of malnutrition

It is clinically useful to categorize the severity of malnutrition depending on the degree of aberration from established thresholds. Suggested phenotypic metrics for grading severity as Stage 1 (moderate) and Stage 2 (severe) malnutrition are shown in Table 4.

### 3.10 Etiology‐based diagnosis classification

An etiology‐based diagnosis classification is endorsed by GLIM consistent with those suggested previously by the International Consensus Guideline Committee,^1^ the AND/ASPEN Guidelines,^7^ and the ESPEN Guidelines.^2^ The classification includes malnutrition related to chronic disease with inflammation, malnutrition related to chronic disease with minimal or no perceived inflammation, malnutrition related to acute disease or injury with severe inflammation, and malnutrition related to starvation including hunger/food shortage associated with socioeconomic or environmental factors (Table 5).

**Table 5:** Diagnosis category according to underlying etiology

Malnutrition related to
• Chronic disease with inflammation • Chronic disease with minimal or no perceived inflammation • Acute disease or injury with severe inflammation • Starvation including hunger/food shortage associated with socio‐economic or environmental factors

## 4 Discussion

This GLIM initiative targets the priority to adopt global consensus criteria so that malnutrition prevalence, interventions, and outcomes may be compared throughout the world. A common malnutrition “language” is a paramount necessity in order to support the development of global standards of care that will promote improved outcomes. The proposed approach for diagnosing malnutrition is based upon a strong consensus endorsing core phenotypic and etiologic criteria that are already in widespread use throughout the world. The intent is to promote global use of these criteria that may in turn be readily used with other approaches and additional criteria of regional preference. The consensus criteria are intended to be simple and readily applied by clinicians and other health practitioners using tools and methods that are readily available. Only modest training should be required. The proposed approach encompasses risk screening and diagnosis but does not entail the robust detail of comprehensive nutrition assessment. It will provide a malnutrition diagnosis that may then be complemented by more comprehensive assessments to provide the basis for individualized care and treatment plans. Consultation of skilled nutrition practitioners like dietitians is recommended for comprehensive assessment based upon regional preferences and availability. Repeated criterion measures over time are recommended so that trajectories of decline, maintenance, and improvement may be identified.

The recommended GLIM approach encompasses both phenotypic and etiologic criteria for the diagnosis of malnutrition but uses only phenotypic criteria cut‐points to provide for severity grading. While etiology has not generally been included in criteria supporting the diagnosis of medical conditions in the ICD construct, the inclusion of etiology has been widely adopted in the clinical nutrition community because it serves to guide appropriate interventions and expected outcomes.^1^ For example, the presence of disease‐associated inflammatory response has potential for major impacts upon treatment approach and anticipated outcome. The GLIM approach acknowledges the diversity and the multi‐factorial etiologies underlying the development of the malnourished phenotype irrespective of body morphology – lean, normal or obese.

Impairment of muscle strength and function are core phenomena in conditions like sarcopenia,^15^, ^16^ cachexia,^5^, ^6^ and frailty.^20^ Assessment of muscle strength should be an integral measure in assessment of patients with suspected sarcopenia since impairment of muscle strength is now recognized as a key component for diagnosis of sarcopenia.^15^, ^16^ Though inflammatory mediators and other mechanisms besides malnutrition are at play, it is recommended that the GLIM consensus criteria be applied to diagnose malnutrition in persons with sarcopenia, cachexia, and frailty so that the priority to undertake appropriate nutrition interventions may be recognized. The most helpful approaches for these conditions will however require combined multimodal interventions beyond nutritional supplements, like pharmacological agents and exercise.

Similarly, patients with cachexia will meet GLIM consensus criteria for malnutrition related to chronic disease with inflammation. Since there is concern that inclusion of cachexia with other disease‐related malnutrition conditions may diminish appreciation for some distinctive features of cachexia, there has been understandable hesitation by some to equate cachexia with this GLIM diagnosis category. The GLIM consensus criteria for malnutrition are therefore intended to be used in parallel with established concepts and nomenclature, including for example, those of cachexia, sarcopenia and frailty.

## 5 Conclusion

A strong GLIM consensus endorsed the selected core phenotypic and etiologic criteria that are already in widespread use throughout the world. Many studies provide clear evidence that the agreed upon criteria for diagnosis of malnutrition are highly relevant and each of them alone is able to predict adverse clinical outcomes. Since these criteria may be readily used with other approaches and additional criteria of regional preference, their global adoption is more likely. As the initiative moves forward the creation of databases that use the selected criteria will facilitate the comparison of malnutrition prevalence, interventions, and outcomes throughout the world. Such observations can be used to support the development of global standards of care that will promote improved outcomes.

After the launch of the GLIM consensus it is important that the nutrition community use the criteria both in prospective and retrospective cohort studies as well as clinical trials in order to validate its relevance for clinical practice. Next steps are to secure endorsements from leading nutrition professional societies and to promote dissemination, validation testing, and feedback. The GLIM consensus should be re‐evaluated based upon review of new studies and advances in screening and assessment every 3–5 years. We will also seek to share the GLIM consensus recommendations with the World Health Organization in the context of the International Classification of Diseases revision process (ICD‐11). This is a high priority since this classification scheme guides clinical diagnosis and reimbursement across much of the world. The proposed GLIM consensus criteria target adults in clinical settings but it will also be a priority to work with the World Health Organization and the United Nations to explore the potential for use in other global settings like famine.

## Conflict of interest

**Gordon L Jensen:** Conflicts of interest/ financial disclosures ‐none.

**Tommy Cederholm:** Conflicts of interest/ financial disclosures ‐none.

**M. Isabel T.D. Correia:** Conflicts of interest/ financial disclosures‐ none.

**M. Christina Gonzalez:** Conflicts of interest/ financial disclosures‐ none.

**Ryoji Fukushima:** Research grant from Taiho Pharmaceutical Factory, Inc.; Honoraria from Otsuka Pharmaceutical Factory, Inc., Terumo Corporation, and Abbotte Japan Co., Ltd.

**Takashi Higashiguchi:** Conflicts of interest/ financial disclosures‐ none.

**Gertrudis Adrianza de Baptista:** Conflicts of interest/ financial disclosures ‐ none.

**Rocco Barazzoni:** Conflicts of interest/ financial disclosures ‐none.

**Renée Blaauw:** Conflicts of interest/ financial disclosures ‐none.

**Andrew JS Coats:** Conflicts of interest/ financial disclosures ‐none.

**Adriana Crivelli:** Conflicts of interest/ financial disclosures ‐none.

**David C Evans:** Paid for consulting by Coram / CVS Infusion (Parenteral Nutr. Advisory Board) and Lyric; Abbott Nutrition and Lyric both paid Evan's institution for research grants; Paid by Abbott Nutrition for speaking honoraria.

**Leah Gramlich:** Conflicts of interest/ financial disclosures ‐none.

**Vanessa Fuchs‐Tarlovsky:** Hospital General de México, Mexico City (honoraria and travel expenses),Tata Memorial Hospital India (travel expenses), UPAEP (Puebla Autonomus University – travel expenses); professional societies including FELANPE, PENSA, the Academy of Nutrition and Dietetics, and the Colegio Mexicano de Nutriologos (travel expenses); and an industry sponsor Fresenius Kabi (travel expenses).

**Heather Keller:** Paid by Nestle Health Sciences for Manuscript focused on dysphagia; Paid for development of educational presentations including service on speakers’ bureaus by Abbott Nutrition and Nestle Health Sciences; Travel/accommodations expenses covered or reimbursed by Abbott Nutrition.

**Luisito Llido:** Conflicts of interest/ financial disclosures ‐ none.

**Ainsley Malone:** Conflicts of interest/ financial disclosures ‐none.

**Kris M Mogensen:** Board membership with ThriveRx; Consultancy from Pfizer, Employment with Brigham and Women's Hospital, Honoraria from Abbott Nutrition Health Institute and Baxter, Royalties from Wolf Rinke Associates.

**John E Morley:** Conflicts of interest/ financial disclosures ‐none.

**Maurizio Muscaritoli:** Conflicts of interest/ financial disclosures‐ none.

**Ibolya Nyulasi:** Conflicts of interest/ financial disclosures ‐none.

**Matthias Pirlich:** Consultancy from seca GmbH, Hamburg, Germany.

**Veeradej Pisprasert:** Conflicts of interest/ financial disclosures‐ none.

**Marian de van der Schueren:** Conflicts of interest/ financial disclosures ‐ none.

**Soranit Siltharm:** Conflicts of interest/ financial disclosures ‐none.

**Pierre Singer:** Grants from Baxter, B Braun, Abbott, and Fresenius Kabi; Honoraria from Baxter, B Braun, Fresenius Kabi, GE, and Cosmed.

**Kelly A. Tappenden:** Conflicts of interest/ financial disclosures ‐none.

**Nicolas Velasco:** Conflicts of interest/ financial disclosures ‐none.

**Preyanuj Yamwong:** Conflicts of interest/ financial disclosures‐ none.

**Jianchun Yu:** Conflicts of interest/ financial disclosures e none.

**Dan L. Waitzberg:** Paid by Fresenius‐Kabi as a member of Member of the Felanpe Award; Paid by Danone for Overview of enteral nutrition in healing manuscript preparation; Travel expenses paid for by Fresenius‐Kabi and Nestle for Aspen and Espen conferences in 2018.

**Andre Van Gossum:** Conflicts of interest/ financial disclosures ‐none.

**Charlene Compher:** Conflicts of interest/ financial disclosures ‐none.

## GLIM Core Leadership Committee

Representing American Society for Parenteral and Enteral Nutrition (ASPEN): Gordon L. Jensen, Charlene Compher

Representing European Society for Clinical Nutrition and Metabolism (ESPEN): Tommy Cederholm, Andre Van Gossum

Representing Federacion Latinoamericana de Terapia Nutricional, Nutricion Clinica Y Metabolismmo (FELANPE): Maria Isabel T.D. Correia, M. Cristina Gonzalez

Representing Parenteral and Enteral Nutrition Society of Asia (PENSA): Ryoji Fukushima, Takashi Higashiguchi.

## GLIM Working Group (alphabetical order)

Baptista G, Barazzoni R, Blaauw R, Coats A, Crivelli A, Evans DC, Gramlich L, Fuchs V, Keller H, Llido L, Malone A, Mogensen KM, Morley JE, Muscaritoli M, Nyulasi I, Pirlich M, Pisprasert V, de van der Schueren MAE, Siltharm S, Singer P, Tappenden K, Velasco N, Waitzberg D, Yamwong P, Yu J.

## Appendix A.

**Table A1:** Cut‐offs suggested in the major screening tools.

	**Phenotypic Criteria**			**Etiologic Criteria**	
	**Weight loss**	**Low body mass index (kg/m** ^**2**^ **)**	**Reduced muscle mass/muscle function**	**Reduced food intake**	**Severe disease/inflammation**
**NRS‐2002** ^12^					
**Mild**	>5% in 3 mo	NS	NA	50–75% of required preceding week	E.g. hip fracture, chronic disease
**Moderate**	>5% in 2 mo	18.5–20.5	NA	25–60% of required preceding week	E.g. major abdominal surgery, stroke
**Severe**	>5% in 1 mo	<18.5	NA	0–25% of required preceding week	E.g. head injury, bone marrow transplantation, intensive care
**MNA‐SF** a ^,21^					
**Mild**	1–3 kg in last months	21–23	NS	NS	NS
**Moderate**	“Does not know”	19–21	“Does not go out”	Moderate loss of appetite past 3 mo	Mild dementia
**Severe**	>3 kg last months	<19	Bed or chair bound	Severe loss of appetite past 3 mo	Acute disease past 3 mo, or severe dementia/depression
**MUST** ^22^					
**Medium risk**	5–10% in 3–6 mo	18.5–20	NA	NS	NA
**High risk**	>10% in 3–6 mo	<18.5	NA	Acute illness AND no food intake for >5 d	NA

a Adapted for older adults (>65 y)

NRS‐2002 = Nutritional Risk Screening‐2002, MNA‐SF = Mini Nutritional Assessment‐Short Form, MUST = Malnutrition Universal Screening Tool, NA = not applicable, NS = not specified

**Table A2:** Cut‐offs suggested in major diagnostic tools for malnutrition and cachexia.

	**Phenotypic Criteria**			**Etiologic Criteria**	
	**Weight loss (%)**	**Low body Mass Index (kg/m** ^**2**^ **)**	**Reduced muscle mass/muscle function**	**Reduced food intake**	**Severe disease/inflammation**
**SGA** ^4^					
**Moderate/Stage B**	5–10% past 6 mo	NA	Mild to moderate deficits in function or muscle mass	“Definite decrease”	Yes
**Severe/Stage C**	>10% past 6 mo	NA	Severe deficit in function and muscle mass	“Severe deficit”	Yes
**Evans 2008** ^5^					
**Cachexia**	>5% in <12 mo	<20	Low FFMI, decreased muscle strength	“Anorexia”	Increased CRP/IL6, low serum albumin (<3.2 g/l)
**PEW 2008** ^23^					
**Protein‐energy wasting**	>5% in 3 mo, or > 10% in >6 mo	<23	Muscle mass down by 5% last 3 mo, or > 10% in >6 mo. Reduced MAC	Energy intake <25 kcal/kg BW/d for >2 mo	Chronic kidney disease, Serum albumin <3.8 g/dl
**Fearon 2011** ^6^					
**Precachexia**	<5%	NA	NA	“Anorexia”	Metabolic change
**Cachexia**	>5% in 6 mo (>2%)	<20 (when WL > 2%)	Sarcopenia ‐ ASMI 7.26/5.45 kg/m^2^ (m/w) when WL >2%	“Often reduced food intake”	Cancer with catabolic drive (systemic inflammation)
**ASPEN/AND 2012** ^7^					
**Moderate**	1–2% in 1 w to 20% in 1 y	NA	Mild muscle loss	<75% of ER for 7 d‐3 mo	Yes
**Severe**	>2% in 1 week to >20% in 1 year	NA	Moderate to severe muscle loss, or reduced grip strength	<50% of ER for >5 d‐ < 1 mo	Yes
**ESPEN 2015** ^8^					
**Malnutrition**	>5% past 3 mo, or > 10%	<18.5, or <20 (<70y)/ <22(>70y)	FFMI <15 kg/m^2^ (f), <17 kg/m^2^ (m)	According to any validated tool	NA

SGA = Subjective Global Assessment, NA = not applicable, NS = not specified, WL = weight loss, PEW = protein energy wasting, MAC = mid‐arm circumference, ASMI = appendicular skeletal muscle index from DEXA, FFMI = fat free mass index
